# Supplementary material for: Exploring empathic engagement in immersive media: An EEG study on mu rhythm suppression in VR
Source: PLoS One. 2024 May 17;19(5):e0303553. doi: 10.1371/journal.pone.0303553 (PMC11101072; doi:10.1371/journal.pone.0303553)
Supplement: Attachment — Submitted filename: Response to Reviewers.docx [file pone.0303553.s001.docx]

Dear reviewers,

We would like to thank you for the detailed and insightful comments. In response, we have made revisions that we feel much strengthened our manuscript. Please see detailed responses to your individual comments below. Much gratitude again for your time and contribution to this work.

**Reviewer #1:** 
Thank you for providing me with this opportunity to review the manuscript titled “Exploring Empathic Engagement in Immersive Media: An EEG study on Mu rhythm suppression in VR”. Hopefully this review statement helps in developing the manuscript further.

To begin with, the introduction could position the manuscript more explicitly vis-à-vis prior literature. For instance, why do we need to compare VR and TV? Furthermore, the argument “empathy…is a crucial element in media experiences” needs more flesh around the bones. How is it a crucial element? What do we know about it so far? In addition, “…for eliciting empathic responses…” (p. 1) would benefit from concrete examples.

In a way, it feels as if the manuscript jumps directly to the topic without properly paving the way. In other words, rushing to the topic results in the manuscript being inadequately positioned with regards to prior literature. As such, the introduction should provide a more nuanced and granulated image of the current body of knowledge.

- We have revised the introduction to include the above comments and minor issues. To avoid abruptly jumping into the topic, we have removed the first paragraph. Originally, our first paragraph was designed to summarize the entire introduction while providing a thesis statement. However, this approach may feel like jumping into the topic too quickly for the readers. Moreover, the subsequent paragraphs have been modified with some additional content to better shape the topic.

Methodology: based on what criteria / prior research were the videos created? Also, were the actors shown in the videos? If so, were they shown in full or partially? Stills of the videos would be very helpful. Also, could you cite similar studies to strengthen the methodologocial choices? What does the simple / complex distinction tell us?

- We referenced several previous studies which have utilized similar methodologies in assessing the activation of the mirror neuron system in action observation or imagery. These studies provided a framework for the complexity levels and types of actions to be included in our videos. The content and references have been added to the text as follows.
  “This grasping movement is a basic action that has been widely employed in several earlier studies (Andric et al., 2013; Di Pellegrino et al., 1992; Muthukumaraswamy et al., 2004; Sartori et al., 2012).”
  “These actions are distinct from simple actions in that they encompass movements of the entire body rather than being confined to a right arm and have two or more interconnected motions. They were adapted from the previous studies investigating how the mirror neuron system is modulated based on the level of complexity of actions (Angelini et al., 2018; Avanzini et al., 2012; Brinkman et al., 2014; Calvo-Merino et al., 2005; Dalla Volta et al., 2015; Sakreida et al., 2005; Yuan et al., 2010).”
- In the videos, actors were shown both in full and partially, depending on the action being performed. For simpler actions such as grasping an object, a closer, more focused shot was used. For complex actions involving whole-body movement (e.g., punching, kicking), a wider shot was employed to capture the entire range of motion. Still images are also added.
- Although object grasping is typically used in action observation studies, its simplicity might limit one’s ability to fully represent empathetic responses. Thus, in this study, by probing the impact of action complexity on these responses, we attempt to indirectly assess the representativeness of this simple task. If the mu rhythm suppression is only triggered by actions in the simple conditions, this could suggest these reactions are specific to certain actions, which may limit our understanding of empathetic responses in more intricate narrative scenarios. Otherwise, the findings might suggest a potential applicability of the study’s results beyond the specific experimental items.
  We do recognise, however, that the complex actions incorporated in this study are not fully representative of the intricate action sequences often found in media narratives. The results of our study may not be directly applicable to scenarios of greater complexity, thus warranting further research into this matter. Nonetheless, we endeavoured to gauge the potential generalisability of this simple material and tried to demonstrate that these responses are not limited to certain types of actions.
  The aforementioned content has been incorporated into both the Introduction and Discussion sections of the document.

Furthermore, engagement towards what? Actor, object, action?

- Due to the ambiguity regarding which part of the text the inquiry addresses, it is difficult for us to provide an accurate answer. Presuming the focus of the query, the participants were instructed to concentrate on the action during observation. This has been reflected in the method section as follows.
  “After agreeing to participate, they were seated in a noise-attenuated shield room. They were then instructed to attentively observe the brief video segments, focusing on the various movements displayed.”

Were the VR and TV clips similar / identical?

- The experimental stimuli comprised 60 short video clips, which were used identically in both media.

Is it possible to share the material so others could replicate your study? Did the author team create the videos or someone else?

- The video was produced by our team, and it can be shared upon request.

Methods section pp. 8-10 does not seem to have any references.

- We added several references regarding materials.

Point being, the methodology section needs to be stronger and more transparent. As it is now, challenging to see how insights were derived from the data.

- In response to your valuable comments, we have tried to enhance the material section to be as transparent as possible. Also, upon request, we will provide all of our materials.

Findings:

Is it possible to have a more nuanced treatment of the findings? For example, were there differences across the videos (not only simple – complex distinction)? As it is now, the findings feel very flat and after reading the manuscript I was hoping to learn more rather than “VR is superior to TV”; yes, I buy this, but how exactly could VR be more superior in this context? Under what conditions? Is VR always more superior?

- We acknowledge that our findings, primarily emphasizing the superiority of VR over TV in eliciting empathetic responses, may appear rather flat. Regrettably, our research did not yield findings beyond these. Although it was not a key element of our research, initially, our hypothesis contemplated the potential for an interaction between complexity and media. However, no significant differences were observed. Ideally, a more nuanced discussion extending beyond the binary classification of simple versus complex would be beneficial. However, the design of our experiment was specifically oriented towards this dichotomy. While the study included different types of actions that might be used as alternative criteria, these were not part of the original experimental design. Therefore, we believe that using them for post hoc analysis would be inappropriate. Given the constraints in extending our findings, we will instead focus on a more comprehensive discussion of the results at hand.

Conversely: my co-author and I studied the use of VR in a public space (metro) and one of our findings was that people felt a bit insecure with a VR headset on. In other words, they weren’t always sure they were safe. While we didn’t compare VR with TV, one could say in public spaces VR might not be as efficient as TV because of the safety / security aspect. So, a more nuanced analysis of the findings would definitely make the paper stronger.

- Your discovery provides us with important implications. Even if users wear a VR headset, if they cannot adequately block out external environments, they will not feel immersed due to a sense of insecurity. Our experiment was conducted in a secure environment, so the participants would not feel unsafe and could be more immersed in the media. This aspect, we believe, is crucial for interpreting our results and has been duly incorporated into our discussion.

For instance, p. 15: “…a more nuanced understanding of empathic engagement dynamics…” – yes, this would be great, yet the manuscript does not address such dynamics at the moment. This would be a great vantage point for making contributions with this study to the current body of knowledge

- We believe that this study enhances existing research by neurophysiologically demonstrating VR’s capacity for eliciting more empathetic responses, not just based on subjective self-reports, thereby contributing to a deeper understanding. This statement was intended to evaluate the contributions of this study while summarizing the paragraph, and it was not meant to invite additional interpretations. The employment of the term ‘dynamics,’ admittedly vague, might have inadvertently suggested the need for additional insights. However, we acknowledge the validity of the reviewer’s critique and recognize the importance of expanding the discourse with further issues that extend beyond our current interpretations. While being cautious about offering interpretations beyond what our results can substantiate, we have composed additional paragraphs in the discussion to enrich it.

Is p. 16 necessary? How does it advance the contributions of the manuscript? Granted, I am definitely not an expert on beta suppression, but regardless this section feels a bit like a sidestep. Instead, I would focus more on the above rather than introducing new concepts.

- This part is not positioned as central to the interpretation of the paper, as pointed out by the reviewer. However, we did find a difference in the simple-complex in the beta band in our results and considered it necessary to present an interpretation of this result. While this might seem somewhat detached from the rest of the findings, we believe it is our responsibility to present an interpretation of the results. Moreover, this finding substantiates the efficacy of our stimuli, demonstrating that the simple and complex stimuli we created were similar but not entirely at the same level and indeed had a discernible impact. We have elaborated on this point, to ensure that this paragraph is integrated into the paper’s comprehensive discussion.

p. 17 – “…VR may evoke stronger empathic responses” – this is super interesting and yet another potentially beneficial vantage point. How could others build on these insights?

Building on the above, and repeated below, it would be great to highlight this vantage point: “how could this paper open up rather than close down discussion?”. How might one build on this work? Instead of reporting findings, present them so that the manuscript explicitly encourages others to do more work in this domain. Here, a helpful vantage point could be this: what might be the three things the reader could take away from this manuscript?

- In response to the insights provided in the aforementioned comments, we have expanded the discussion section by incorporating two additional paragraphs. We appreciate your valuable feedback.

Once again, thank you for the opportunity to review this manuscript. Hope this review statement helps in developing the manuscript further.

Minor comments:

- After revising the manuscript, the abstract could also be revised so it does not oversell the findings. There is no need for that. Instead, focus on the contributions and how these findings open up rather than close down discussion.

- P. 3 – “Empathy, encompassing both…” sentence repeats what has been said above
- P. 3 – last paragraph is very interesting and promising
- P. 4 – “Immersion captivates…” – could be rephrased; how exactly does immersion lead to emotional connection?
- P. 4 – “For instance, in cinema…” – not sure the causality presented in this sentence is clear. Either tone down or explicitly explain the situation to the reader
- P. 4 – top paragraph – in general, the top part presents strong causalities that, in fact, are not necessary from the paper’s contributions’ point of view. Instead, provide a more nuanced treatment of the current body of knowledge so the paper’s contributions stand out on their own.
- P. 5 “ultimate empathy machine” – similarly, a very strong statement. No need to oversell the topic.
- P. 5 – Clouds Over Sidra – this is a good example, yes, but are there more examples?
- P. 5 middle paragraph is very nice as it has a nuanced and granular take on prior literature
- P. 6 – top paragraph – examples would make this paragraph more accessible
- P. 17 – “…superiority of VR…” – yet another strong statement

- Thank you very much once again for your thoughtful comments on the paper. Your feedback was invaluable for refining the study and ensuring a more nuanced understanding of the research outcomes.

**Reviewer #2:**

The manuscript is well-organized and clearly written, with a well-defined research question and hypothesis. The methods are rigorous and appropriate for addressing the research question, and the results are clearly presented and supported by the data. I particularly appreciate the thoroughness of the discussion section, which provides a comprehensive analysis of the findings and their implications.
Overall, I believe that the manuscript is a valuable contribution to the field, and I am confident that it will be well-received by the readership of PLOS ONE. I look forward to seeing it published.

- We really appreciate your thoughtful review and positive feedback on the manuscript. It's great to hear that the research question and hypothesis were well-defined, and the methods, results and discussion met the standards for rigor. Your positive feedback on the manuscript is encouraging. Once again, thank you very much for your time and contribution.

**Reviewer #3:**

The introduction effectively contextualizes the research, outlines the research questions, highlights the significance of the study, and provides a clear hypothesis. It also demonstrates a solid connection to existing literature and a comprehensive understanding of the topic, making it a well-structured and informative paper section.

The front matter provides a clear and informative background on immersive media technologies, especially virtual reality (VR), and their potential impact on empathy. It effectively sets the stage for the research study by outlining the significance of empathy in media experiences. The paper includes citations from prominent media studies and empathy scholars; therefore, the research is grounded in existing literature and theoretical frameworks.

The paper effectively highlights a research gap related to the effectiveness of VR in inducing empathy and the lack of neurophysiological evidence in this domain. It then articulates the central research questions. The discussion on the relationship between immersion and empathy is well-founded and coherent. It explains how immersion in various media forms can foster empathic engagement and sets the stage for the unique immersive qualities of VR.

The authors discuss the distinctive characteristics of VR, such as its capacity to create a sense of presence and interactivity, effectively underscores why VR is of particular interest in the study of empathy and cites previous research to support claims about VR's immersive qualities — adding credibility to the later discussion. The paper acknowledges the existence of mixed results in earlier research regarding VR's impact on empathy — demonstrating an awareness of the complexity of the topic and a willingness to critically assess the claims made about VR as the "ultimate empathy machine."

The introduction proposes the neurobiological basis of empathy and its connection to the mirror neuron system — adding depth to the discussion and positioning the research within the field of neuroscience. The explanation of the mu rhythm and its connection to empathic engagement is well-defined. It explains how EEG data can be used as an empirical measure of empathy, a vital aspect of the study's methodology.

The introduction concludes with a clear hypothesis that states the expected outcome of the study, which is that VR will lead to a more robust empathic response compared to TV and that the complexity of actions may modulate this response.

The Methods section outlines the comprehensive and well-structured research design, data collection, and analysis procedures. The section begins with a clear and concise description of the participants, including their demographics and the recruitment method. Including gender and age information and the number of participants is crucial for transparency. Mentioning that the experiment was conducted per the university's ethics guidelines reinforces the ethical considerations.

The description of the experimental stimuli is detailed, including information about the camera used and the source of the actors. Categorizing stimuli into simple and complex actions adds clarity to the design. However, providing information on the number of clips in each category would be beneficial to understanding the balance in stimuli representation.

The procedure section offers a step-by-step explanation of how the experiment was conducted. It covers participant instructions, EEG data collection, and the overall time frame of the investigation. The random assignment of participants to TV and VR conditions and the provision of breaks between states is essential for minimizing order effects and ensuring participant comfort. The compensation details are also evident.

The description of the equipment used for the TV and VR conditions is detailed and adequately explains how the VR headset and EEG devices were combined without interference — this is vital information for understanding how the experiment was conducted. The section clearly describes the EEG recording setup, including the type of EEG recorder, electrode placement, and electrode reference. The explanation of how the VR headset was combined with EEG devices is well-detailed and suggests care in the experimental setup.

The preprocessing steps are thoroughly explained, from filtering to artifact rejection. Including the Python packages and the description of the data loss due to artifact rejection adds transparency to the methodology. However, mentioning the specific criteria for data rejection might be helpful to address potential concerns about subjective judgments.

The description of the time-frequency analysis is concise and mentions the choice of electrodes, frequency range, and power normalization. These details provide insight into the analytical approach. The section explains the statistical methods employed, including repeated measures ANOVA and non-parametric permutation F-test. It mentions the handling of multiple comparisons and correction methods, enhancing the rigor of the analysis.

The Results section presents the findings, including the statistical tests performed and the graphical representations of the data. The area begins with the results of the repeated measures ANOVA, which is appropriate for testing main effects and interactions. The main impact of media on both C3 and C4 channels is significant, with p-values provided — this demonstrates that the media condition (VR vs. TV) has a notable impact on mu rhythm suppression in the specified EEG channels. The significance level is appropriately indicated (p < 0.001). The results effectively demonstrate that the results from the non-parametric permutation F-test are consistent with the ANOVA findings — this adds credibility to the results by showing consistency across two statistical methods. The report also notes the absence of significant clusters in the complexity analysis, which is essential information for the reader. It highlights that no significant differences in mu rhythm suppression were found between simple and complex conditions. The significance threshold (p<0.025) is clearly stated. The inclusion of figures (Figure 1, Figure 2, and Figure 3) helps visualize the results. Figure 1 provides a graphical representation of the mu rhythm suppression across conditions, assisting the readers to interpret the main effect of media and complexity. Figure 2 offers power spectrograms, which help understand changes in power across the frequency range. Figure 3 includes a clear and informative legend that explains the color coding used to indicate significant clusters. The distinction between blue and red shading for more significant suppression or enhancement is presented, making the results easier to interpret. Still, the color coding needs to be changed for color-blind accessibility.

- We thank you for pointing this out. The figures’ colors have been edited for color blind accessibility, avoided using red and green.

The paper's Discussion section effectively addresses the findings and provides insightful interpretations and implications. The section provides a clear and comprehensive understanding of the study's results. It appropriately discusses the main conclusions of mu rhythm suppression in VR versus TV conditions. The discussion effectively links the results to previous immersive media, empathy, and mirror neuron system activation research. This connection to existing literature adds depth and context to the findings. The paper correctly highlights the advantages of using EEG to measure neural responses. The discussion points out the benefits of objective, neurophysiological measures in contrast to subjective self-report measures often used in previous studies. The section addresses the role of action complexity in the findings and raises valuable points about the limitations of using simple actions in the study. The discussion acknowledges the potential limitations and opens the door for future research.

The discussion of beta suppression and its potential implications for the observed differences between simple and complex conditions is well-presented. It appropriately suggests that future research should delve deeper into the influence of action complexity on neural responses. This section successfully summarizes the study's main contributions to the field, emphasizing the potential of VR as an empathy-enhancing medium. It highlights the consistent mu rhythm suppression observed in VR and the need for further research to explore different media types and actions.

The paper concludes with insightful suggestions for future research, including exploring action complexity, the role of media immersion in empathic engagement, and potential applications in various domains. The final section is well-structured and effectively interprets the results in the context of existing literature. It also identifies limitations and provides valuable directions for future research, making it a solid and informative part of the paper.

The paper demonstrates a clear and well-structured format, making it easy for readers to follow the research design, methods, findings, and interpretations. The article effectively integrates relevant literature into the introduction and discussion sections, enhancing the study's context and relevance. Using EEG as a neurophysiological measure is appropriate, and the paper provides a good rationale for this choice. It highlights the advantages of using objective criteria compared to subjective self-report measures. The report explores an exciting and relevant topic by investigating immersive media's impact on empathic experiences, particularly VR. This research area has practical applications and implications for various fields. The methods section is well-detailed, and using both ANOVA and non-parametric permutation F-tests provides robust statistical analysis. The paper addresses participant demographics, materials, apparatus, and data analysis factors. The "Discussion" section is a strength of the article. It effectively interprets the results, connects them to existing literature, acknowledges limitations, and suggests promising directions for future research.

While the paper demonstrates several strengths, some areas could be improved or have limitations.
• The sample size in the study is relatively small (30 participants), which may limit the generalizability of the findings. Increasing the sample size could strengthen the study's reliability and validity.

- Thank you for highlighting the concern about the sample size in the study. We agree that a larger sample size would undoubtedly enhance the study's reliability and validity. We will carefully evaluate the feasibility of expanding the sample size in future iterations of the research.

• The study mentions recruiting university students, which might introduce a potential bias in the sample's demographic characteristics. More information on participant demographics, such as age, background, and experience with VR, could be helpful.

- Thank you for pointing out the potential bias in the sample's demographic characteristics, specifically the recruitment of university students. As your suggestion to include more detailed participant demographics, such as background, and experience with VR, we addressed this in the revised version of the manuscript, providing a more comprehensive overview of the sample characteristics.

• The paper does not mention diversity in the sample, which is a common concern in research. It's essential to consider the impact of gender, age, and cultural background on empathic responses.

- Thank you for highlighting the concern about the lack of information on diversity in the sample. We acknowledged the importance of considering gender, age, and cultural background in relation to empathic responses. We have provided a more thorough examination of these demographic factors in the manuscript, addressing the potential impact on the study's findings.

• The paper acknowledges the limitation of using simple actions for the study. Exploring a broader range of activities, including those more typical of complex media narratives, would be valuable — for example, the 2021 paper Exploring Virtual Reality for Quality Immersive Empathy Building Experiences in Behaviour & Information Technology.

- Thank you for your comment on the limitation of using simple actions in the study, suggest exploring a broader range of activities, particularly those typical of complex media narratives. We agree with the importance of diversifying the activities to capture a more comprehensive understanding of empathic responses. However, in this study, in order to eliminate the variables arising from this, only very simple scenes(for example, )were produced and used as stimuli. We will consider and incorporate this suggestion in future iterations of the study, exploring a wider range of actions. Additionally, we reviewed the research, "Exploring Virtual Reality for Quality Immersive Empathy Building Experiences in Behavior & Information Technology," to gather insights for enhancing the study's design. We thank you for your valuable suggestions.

• The study's findings are based on specific experimental conditions, and the paper acknowledges that more research is needed. It's important to highlight that the results may not universally apply to all VR or media content.

- We thank you for your attention to the study's acknowledgment of the need for more research and the specific experimental conditions. We completely agree with your point about emphasizing that the study's findings may not be universally applicable to all VR or media content. We made sure to underscore this limitation more explicitly in the paper to provide a clear context for the results.

• The study is conducted in a controlled laboratory setting, and the actions in the videos are artificial. It may not fully represent the complexity and emotional engagement of real-world media experiences.

- Thank you for your observation regarding the study’s-controlled laboratory setting and the artificial nature of the actions in the videos, acknowledgment of the potential limitations in capturing the complexity and emotional engagement of real-world media experiences. We have addressed some of this concern explicitly in the discussion section of the manuscript, discussing the implications of the controlled setting on the study's external validity.

• While the study looks at neural markers associated with empathy, it's essential to recognize that empathy is a complex psychological and neurobiological phenomenon. The paper acknowledges this but should emphasize the complexity of the topic further.

- Thank you for this comment and the opportunity to provide an additional explanation. We agree that it's important to further emphasize the multifaceted nature of empathy, both psychologically and neurobiologically. In the revised version of the manuscript, we provided a more discussion on the intricate aspects of empathy, acknowledging its complexity and the various factors that contribute to its understanding.

• The paper mentions obtaining informed consent and following ethical guidelines. Still, more information on the ethical aspects of the research, particularly in the context of using VR technology, would be beneficial.

- Thank you for your feedback regarding the ethical aspects of the research, especially in the context of using VR technology. In the revised version of the manuscript, we included discussing issues related to participant consent, potential risks, and the overall ethical guidelines followed in the study.

• The paper's conclusion could be more concise and emphasize the essential findings and implications.

- Thank you for your suggestion to make conclusion more concise and emphasize the essential findings and implications. We have refined the conclusion to ensure a more focused summary of the study's key outcomes and their broader significance.

It's important to note that many of these concerns are typical of scientific research and should be considered opportunities for further investigation and improvement rather than significant flaws.

In summary, the paper demonstrates a robust research design, a clear presentation of results, and insightful interpretations. It effectively contributes to understanding the relationship between immersive media, empathy, and neural mechanisms. Overall, the paper appears to be of good quality and contributes meaningfully to its field of study.

- I'm grateful for your positive assessment of the paper. It's rewarding to know that the research design, results presentation, and interpretations were effective in conveying the study's findings. I'm particularly pleased to hear that you believe the paper makes a meaningful contribution to the field of study. Thank you for taking the time to provide your feedback.

***We have so far tried our best to revise our manuscript and clarify important concerns that you raised. Please do not hesitate to let us know if you need more information or have other questions or concerns. Thank you very much again, for your time and contribution.
