# Supplementary material for: Exploring empathic engagement in immersive media: An EEG study on mu rhythm suppression in VR
Source: PLoS One. 2024 May 17;19(5):e0303553. doi: 10.1371/journal.pone.0303553 (PMC11101072; doi:10.1371/journal.pone.0303553)
Supplement: Attachment — Submitted filename: Response to Reviewers (Minor Revision).docx [file pone.0303553.s002.docx]

***Dear reviewers,***

***We would like to thank you once again for the detailed and insightful comments. We have so far tried our best to revise our manuscript and clarify important concerns that you raised. Please see detailed responses to your individual comments below. Much gratitude again for your time and contribution to this work.***

Reviewer #1

Introduction (minor issue): while this section has considerably improved, there is room for making it even stronger to help the readers engage with the manuscript more thoroughly. For instance, this is how the introduction’s structure could look like:

1. Engagement has been found to be an issue in immersive media

2. Empathy, amongst other factors such as immersion, plays a crucial role in evoking engagement

3. Yet, we do not know much about how, and through what mechanisms, VR evokes empathic engagement

4. Thus, more research is required to better understand how empathic engagement in VR differs from more traditional media

The above is just a suggestion, mind you. The rest of the manuscript is now very strong, and getting the introduction to the same level would be very critical at the moment. To be precise, I do not see this as a major undertaking; all the key ideas are there in the introduction, it just needs a stronger structure.

*➔ We thank you for pointing this out. We have further revised the introduction based on your suggestions, focusing on the relationship between empathy and engagement in the first paragraph.*

Methodology (tiny comment): Were the participants given USD 24 for their participation or X won equivalent to USD 24? (Also, “$24 USD” should be either $24 or USD 24) As said, tiny comment, but crucial in the sense of ensuring the manuscript does not contain any ambiguities. As a concrete suggestion, I would disclose how many won each participant received and in brackets how much it would be in USD. This, of course, assuming that the participants received won instead of USD. If they did receive USD, my apologies for the wrong assumption. In such case, please only consider the comment on the spelling format.

*➔ Thank you for providing comments down to the tiny details that we had overlooked. We have revised the procedure based on your suggestions: “Participants were compensated with 30,000 KRW (equivalent to approximately 24 USD) for their participation.”*

Contributions (minor issue): at the end of the discussion, I would highlight the contributions more explicitly from two perspectives: 1) how do the findings contribute to extant body of knowledge, and 2) how do the findings open up rather than close down conversation (i.e. future research avenues). Again, all the information is there, I would just make these two aspects very explicit to the reader so that the manuscript would receive the attention and engagement it deserves.

*➔ In the conclusion section, we aimed to explicitly mention two perspectives you highlighted. Thank you once again for guiding us toward making a more substantial contribution to the field.*

Reviewer #3

The discussion provides a thoughtful interpretation of the findings, linking them to existing research and theory. It also acknowledges the study's limitations and suggests avenues for future research, demonstrating the authors' critical engagement with their work. **The paper could benefit from a broader discussion on the generalizability of its findings. For example, the sample size and demographics (e.g., all participants being university students) may limit the applicability of the results to broader populations.**

*➔ We appreciate insightful feedback highlighting the importance of addressing the generalizability of our findings We acknowledge that our current results, obtained from experiments conducted in a laboratory setting with college student participants, may not necessarily apply to a broader population. Our experimental limitations are similar to those in typical experimental studies and are not particularly unique. We added suggest directions for future research to explore these findings in more diverse populations, thereby enhancing the understanding of our study's implications across different demographic groups.*

**While the paper posits that the immersive quality of VR is primarily responsible for enhanced empathic responses, it would be beneficial to consider and discuss alternative explanations or contributing factors more thoroughly. For instance, the role of narrative engagement, user interactivity, and the novelty of VR technology could also influence empathic responses.**

*➔ The aspects you mentioned, especially user interactivity, could have a significant impact on empathy. Our experiment, however, did not investigate this factor and primarily concentrated on isolation. In light of your feedback, in the future research, we will develop the research to incorporate these aspects more thoroughly, providing a more holistic view of how VR can enhance empathic responses. We believe that by exploring these additional factors, our paper will offer a more comprehensive understanding of the potential of VR in empathy-related applications. Thank you very much for guiding us toward making a more substantial contribution to the field.*

****Thank you very much once again for your thoughtful comments on the paper. Your feedback was invaluable for refining the study and ensuring a more nuanced understanding of the research outcomes.***
